# Tannins from *Acacia mearnsii* De Wild. Bark: Tannin Determination and Biological Activities

**DOI:** 10.3390/molecules23040837

**Published:** 2018-04-05

**Authors:** Sosuke Ogawa, Yoshikazu Yazaki

**Affiliations:** 1 Mimozax Co., Ltd., 4291-1, Miyauchi, Hatsukaichi-shi, Hiroshima 738-0034, Japan; 2 Department of Chemical Engineering, Monash University, Clayton, Victoria 3800, Australia; yoshi.yazaki@monash.edu

**Keywords:** *Acacia mearnsii* bark, wattle tannin, proanthocyanidins, biological activities

## Abstract

The bark of *Acacia mearnsii* De Wild. (black wattle) contains significant amounts of water-soluble components acalled “wattle tannin”. Following the discovery of its strong antioxidant activity, a wattle tannin dietary supplement has been developed and as part of developing new dietary supplements, a literature search was conducted using the SciFinder data base for “*Acacia* species and their biological activities”. An analysis of the references found indicated that the name of *Acacia nilotica* had been changed to *Vachellia nilotica*, even though the name of the genus *Acacia* originated from its original name. This review briefly describes why and how the name of *A. nilotica* changed. Tannin has been analyzed using the Stiasny method when the tannin is used to make adhesives and the hide-powder method is used when the tannin is to be used for leather tanning. A simple UV method is also able to be used to estimate the values for both adhesives and leather tanning applications. The tannin content in bark can also be estimated using NIR and NMR. Tannin content estimations using pyrolysis/GC, electrospray mass spectrometry and quantitative ^31^P-NMR analyses have also been described. Tannins consists mostly of polyflavanoids and all the compounds isolated have been updated. Antioxidant activities of the tannin relating to anti-tumor properties, the viability of human neuroblastoma SH-SY5Y cells and also anti-hypertensive effects have been studied. The antioxidant activity of proanthocyanidins was found to be higher than that of flavan-3-ol monomers. A total of fourteen papers and two patents reported the antimicrobial activities of wattle tannin. Bacteria were more susceptible to the tannins than the fungal strains tested. Several bacteria were inhibited by the extract from *A. mearnsii* bark. The growth inhibition mechanisms of *E. coli* were investigated. An interaction between extracts from *A. mearnsii* bark and antibiotics has also been studied. The extracts from *A. mearnsii* bark inhibit the growth of cyanobacteria. Wattle tannin has the ability to inactivate α-amylase, lipase and glucosidase. *In vivo* experiments on anti-obesity and anti-diabetes were also reported. Several patents relating to these enzymes for anti-diabetes and anti-obesity are in the literature. In addition, studies on *Acacia* bark extract regarding its antitermite activities, inhibition of itching in atopic dermatitis and anti-inflammatory effects have also been reported. The growth of bacteria was inhibited by the extract from *A. mearnsii* bark, and typical intestinal bacteria such as *E. coli*, *K. pneumoniae*, *P. vulgaris* and *S. marcescenes* was also inhibited in vitro by extracts. Based on these results, the *Acacia* bark extract may inhibit not only the growth of these typical intestinal bacteria but also the growth of other types of intestinal bacteria such as *Clostridium* and *Bacteroides*, a so-called “bad bacteria”. If the tannin extract from *A. mearnsii* bark inhibits growth of these “bad bacteria” *in vivo* evaluation, the extracts might be usable as a new dietary supplement, which could control the human intestinal microbiome to keep the body healthy.

## 1. Introduction

The bark of *Acacia mearnsii* De Wild. (black wattle) contains significant amounts of water-soluble components, known as “wattle tannin”, which has been used for producing tanned leather for more than one hundred years and for the manufacture of water-resistant and structural wood adhesives for more than fifty years. Based on the initial discovery of the extremely high superoxide scavenging activity (SOSA) of the hot water extract from the bark of *A. mearnsii* De Wild. in 2002 and following a series of toxicity, safety and biological tests of the wattle tannin, including inhibition of lipase and α-amylase, a wattle tannin dietary supplement was developed in 2007. Since then, a supplement for human health has been marketed as ACAPOLIA^®^ in Japan [1].

In order to explore additional possibilities for the use of wattle tannin in dietary supplements, a literature search using the SciFinder database (produced by Chemical Abstracts Service) was undertaken. A total of 489 references was found when the keywords “*Acacia*” and “biological activity” were searched (search performed on 13 June 2017). After a careful reading of those references on *Acacia* species and their biological activities, an important event was recognized. In spite of the fact that the name of the genus *Acacia* was derived from the name of *Acacia nilotica*, in 2013 the name of this particular plant was changed to *Vachellia nilotica*. This name change is critical for anyone searching the literature for the biological properties of *A. nilotica* where apparently there has been nothing reported after 2013. The new *V. nilotica* species name must be used to obtain data published after 2013. Consequently, a literature search (performed on 29 August 2017) on *A. mearnsii* as a keyword was conducted resulting in a total of 369 found references. This review briefly describes the historical events of the species name change of *A. nilotica* and then the determinations of wattle tannin from *A. mearnsii* bark and its biological activities with a view to developing new dietary supplements.

## 2. Taxonomy of *Acacia*

### 2.1. Acacia Mearnsii and Acacia Nilotica (Origin of the Genus “Acacia”)

*A. mearnsii* De Wild. (black wattle) belongs to the family Fabaceae (pea family) and is a fast-growing native tree, which occurs naturally in south-eastern Australia. The genus *Acacia* contains over 1000 species, just behind the largest genus in the Fabaceae family, *Astragalus* which contains over 3000 species [2].

According to Kew*science*, Plants of the World online [3], *A. nilotica* (now *V. nilotica*) was used in medicine during the early Egyptian dynasties some 3500 years ago. Pedanius Dioscorides (a Greek physician and ‘father of botany’, ca. 40 to 90 A.D.) described a preparation of extracts from the leaves and fruit pods of a plant in his book on “Medicinal Material” and he called it ‘akakia’, from which the name of the genus *Acacia* originated. *Acacia* was formally adopted by Miller [4] in a paper describing 24 African and American species. However, his generic concepts were so broad that a number of his species are no longer accepted as belonging to the genus *Acacia.* Prior to Miller, the name *Acacia* had been widely used in pre-Linnean literature [5]. Linnaeus [6] had placed 39 species in the genus *Mimosa* and two of these species were *Mimosa scorpioides* and *Mimosa nilotica*; these taxa were subsequently transferred to *Acacia* and *Acacia scorpioides* is now considered to be conspecific with *A. nilotica* [7].

There are some 1350 species of *Acacia* found throughout the world and approximately 1000 species found in Australia, where it is commonly known as “wattle”. *Acacia* is the largest genus of vascular plants in Australia [8]. Based on morphological, palynological and biochemical characteristics, Pedley [9] in 1986 proposed that the genus *Acacia* could be divided into three genera: *Acacia* (161 species), *Senegalia* (231 species) and *Racosperma* (960 species). Although *A. nilotica* belongs to the smallest genus *Acacia*, since it was not only native to Africa but also named as the first *Acacia* species, South Africans believed that the name of “*Acacia*” should be retained to include *A. nilotica*. In contrast to this view, there are 960 species belonging to the genus *Racosperma* and most species are found in Australia. Australians believed wattles (*Acacia* species) to be an Australian icon, so that a proposal to keep the name *Acacia* instead of *Racosperma* for the Australian *Acacia* plants was made in 2003 [7,10]. The proposal was accepted at the 17th International Botanical Congress (IBC) in Vienna in July 2005. However, since then, argument and controversy on the change of genus name *Acacia* had not diminished and many arguments between African and Australian scholars have occurred [11]. In order to resolve this problem at the 18th IBC in Melbourne in July 2011, prior to the Congress, eight distinguished taxonomists published their pragmatic view on retypification of *Acacia* Mill. with an Australian type in Taxon [12] and finally the argument reached the conclusion that the proposed change in type and use of the name *Acacia* only for the Australian species were approved at the 18th IBC in Melbourne in July 2011. Following the IBC, South African scientists conducted phylogenetic studies on *Acacia sensu lato* including approximately 140 African species using details of the morphology and DNA sequence data. As a result from this study, two generic names were recognized as being able to accommodate the African taxa, namely *Senegalia* and *Vachellia*, in which the former *A. nilotica* is now named *V. nilotica*. This was due to the differences in not only taxonomical and phytochemical characteristics but also more decisively to differences in the genetic DNA sequences [13]. Tannin from the bark of the formerly known *A. nilotica* belongs to the hydrolysable tannins class, whilst tannin from *A. mearnsii* bark belongs to the condensed tannins [14]. New names for the African *Acacia* species were officially announced in 2014 [15,16].

### 2.2. Acacia Mearnsii De Wild.

The Belgian naturalist Émile Auguste Joseph De Wildeman first described *A. mearnsii* De Wild. in 1925 [17]. This species was first collected by E.A. Mearns from a cultivated specimen in East Africa [18]. *A. mearnsii* is native to south-eastern Australia and Tasmania, naturalized in Western Australia, India and the Hawaiian Islands and introduced into Africa, the Caribbean, East Asia, Europe, Sri Lanka, North America, New Zealand, South America and Southeast Asia [19]. *A. mearnsii* plantations covering ca. 300,000 ha have been established in South Africa, Brazil, China and Vietnam [20,21]. The bark of *A. mearnsii* contains significant amounts of water-soluble “wattle tannin” as has been noted previously [1].

## 3. Analyses Method of Wattle Tannin and Their Composition

### 3.1. Wattle Tannin Extracts and Tannin Analyses

Wattle tannins are composed of polyflavanoids and their precursors, which are the major components, together with other phenolic components and complex mixtures of carbohydrate gums, sugars and amino acids. Polyflavanoids consist of a large number of individual components, whose molecular masses may range from 300 for the monomeric compounds up to 3000 for the large polymers. Two methods of estimating the tannin contents in a sample are the Stiasny method and the hide-powder method. The principle of the Stiasny method is to estimate the amount of the polyflavanoid components in tannin extracts which react with formaldehyde in acid solution. This method is employed particularly when the tannin is used in the production of wood adhesives. The hide-powder method is based on the affinity of tannin components towards collagen. However, the true affinity of flavonoid compounds to proteins only becomes apparent from the triflavanoid level indicating that low molecular weight polyflavanoids with a molecular mass less than approximately 800 may not react with protein [22]. The hide-powder method is used for the application of wattle tannin to leather tanning.

#### 3.1.1. Polyflavanoid Contents Analyzed Using the Stiasny Method for Wood Adhesives

The yield and quality of wattle tannin from a bark are economically extremely important. *A. mearnsii* was introduced into China in the 1950s, but was identified by the Chinese government as a promising species for tannin production only in early 1980. In 1985, the Australian Centre for International Agricultural Research (ACIAR) commenced research collaboration with the Chinese Academy of Forestry (CAF). One of the aims was to identify well-adapted, high tannin yielding provenances of *A. mearnsii* in Australia. Special seed collections of *A. mearnsii* were made in Australia and at the same time bark samples from the twenty provenances were collected and the yield of tannin extract and polyflavanoids content for the application to wood adhesives were analyzed using the Stiasny method. Results from these analyses showed that the barks of *A. mearnsii* trees from samples provenances in both Victoria and Tasmania contained higher yield tannin and polyflavanoids than provenances in either New South Wales or South Australia [23].

#### 3.1.2. Molecular Size Analysis of Wattle Tannin Extracts Using Ultrafiltration

Great variability in the growth rate, flowering periods, thickness of bark, the extent of gummosis and disease resistance appears among *A. mearnsii* trees in China. In addition, the quality of the extracts can be variable. In order to overcome the variable quality of extracts and also to assess the quality of extracts, an ultrafiltration method was developed, particularly for the application to wood adhesives. Two tannin extracts were commercially produced in China and South Africa and the aqueous (90 °C) extract of the barks from *A. mearnsii* trees (2–5 years old) was prepared at the CAF laboratory in Nanjing, China. On the basis of purity of polyflavanoids and also the preliminary viscosity for these wattle tannins, wattle tannin extracts from *A. mearnsii* bark in China can become a potential basis for wood adhesives production [24].

#### 3.1.3. Tannin Content of Wattle Tannin Using Hide-Powder Method for Leather Tanning

In the middle 1980s in China, most of the tannin extracts obtained from barks of *Larix* and *Pinus* species had been used as tanning agents by leather manufacturers. The hide-powder method had been used for tannin analysis in the leather tanning industry [25] but it is labor intensive, whilst the Stiasny method is regarded as a rapid and reliable method. Therefore, the tannin contents of the bark samples from 18 of the 20 provenances were determined using both the hide-powder and the Stiasny methods. The results from the statistical analyses showed that although the correlation between tannin contents in the total solids and Stiasny values was marginally significant at *p* = 0.05, the Stiasny values were not able to be used to predict tannin contents for the treatment of leather [26].

#### 3.1.4. Tannin Analyses Using UV, Stiasny and Hide-Powder Methods

Although the major components of wattle tannin extracts are polyflavanoids, which show strong absorption in the ultraviolet (UV) region at 250–280 nm, other phenolic compounds, which do not react with formaldehyde and/or protein also show strong absorptions in this UV region. Roux [27] developed a simple and extremely rapid UV method for tannin analysis for leather tannin. Consequently, the UV method was used to determine tannin contents of wattle tannin extracts which had been analyzed previously by both the Stiasny and the hide-powder methods. In addition, the relationships among the results obtained by these three methods were statistically analyzed with a desire to replace the laborious and time-consuming hide-powder and the Stiasny methods by the more rapid UV method. A Chinese wattle tannin, which had been previously analyzed [27] was fractionated into six fractions using an ultrafiltration technique and each fraction was analyzed by the hide-powder, the Stiasny and the UV methods. Since the ethyl acetate soluble fraction gave the highest values by both the Stiasny and the hide-powder methods, this was regarded as a standard tannin fraction. Consequently, these values (i.e., 108.9, 120.4 and 93.0 for the Stiasny, the UV and the hide-powder method, respectively) were used as standards for the three methods. The original values of wattle tannin were 95.4%, 105.3% and 81.1% for the Stiasny, the hide-powder and the UV methods were divided by these standard values, respectively, so that tannin contents calculated were 87.6, 87.5 and 87.2, respectively. Thus, the UV method is a quick and simple procedure which can be used to estimate both Stiasny values for wood adhesives and tannin contents for leather tanning [28].

### 3.2. Estimation of Tannin Contents in the Bark Using NIR and NMR

Near infrared (NIR) spectroscopy was previously investigated as an alternative to the traditional methods of bark analysis for *A. mearnsii* [29]. The availability of the bark samples studied together with the data obtained from the analyses using the hide-powder, the Stiasny and the UV methods provided an opportunity in investigate whether NIR spectroscopy could be used to estimate several parameters in a set of *A. mearnsii* bark samples. The analysis of two sets of *A. mearnsii* De Wild. samples by NIR spectroscopy were studied. Set 1 samples were characterized in terms of hot water extractives, Stiasny value and polyflavanoid content, whilst Set 2 samples were characterized by nine different parameters, including tannin content. Calibrations developed for hot water extractives and polyflavanoid content (Set 1) gave very good coefficients of determination and performed well in prediction. Set 2 calibrations were generally good with total and soluble solids, tannin content, Stiasny value-2 and UV-2. However, owing to the small number of Set 2 samples, no predictions were able to be made using the calibrations. The study concluded that NIR spectroscopy had considerable potential for the rapid assessment of the quality of extractives in *A. mearnsii* bark [30].

In order to obtain a direct estimation of the tannin content in *A. mearnsii* bark, the application of NIR spectroscopy together with multivariate calibration methods were studied on samples of barks which were natural non-treated, and also which had been dried and milled. Ten determinations per hour including the sample preparation procedures were claimed to be able to be completed using NIR with a time of twenty hours for each determination using the Standard (NBR 11131) method [31]. The NIR method has been further developed [32].

An analytical method based on the solid state ^13^C-NMR spectrum of bark [33] has been reported. The solid state ^13^C-NMR of ground *A. mearnsii* bark before and after tannin extraction were obtained. The signal intensities were normalized against the 173 ppm hemicellulose signal based on the assumption that hemicelluloses were not extracted. At least ca. 80% of the total tannin was extracted from the *A. mearnsii* bark. Thus, solid state ^13^C-NMR offers the advantage of being applicable to source materials in their native state, and has potential applications in optimizing extraction processes, identification of tannin sources, and characterization of tannin content in cultivar yield improvement programs. 

### 3.3. Proanthocyanidin Composition of Wattle Tannin from A. mearnsii Bark

Wattle tannin has been commercially produced by extracting *A. mearnsii* bark with hot water. The major components of wattle tannin are “condensed tannin” which consists of flavanoid units (mainly flavan-3-ols) condensed to varying degrees. The distinctive flavan-3-ols are fisetinidol, robinetinidol, catechin and gallocatechin. These flavanoid monomers are attached to one another by means of carbon-carbon linkages, so that polymeric flavonoids are formed by the 4-8 and the 4-6 bonds and four biflavanoids: fisetinidol-(4α-8)-catechin, robinetinidol-(4α-8)-catechin and robinetinidol-(4α-8)-gallocatechin (all trans stereochemistry) and fisetinidol-(4β-8)-catechin (2,3-*trans*-, 3,4-*cis*: 2′,3′-*trans*-stereochemistry) [34] and two triflavanoids: robinetinidol-(4α-8″)-robinetinidol (4′α-6″)-gallocatechin and robinetinidol-(4α-8″)-robinetinidol (4′α-6″)-catehin have been isolated and identified [35]. A study on wattle tannin from *A. mearnsii* bark in China isolated and identified three dimeric proanthocyanidins: robinetinidol-(4α-8)-catechin, fisetinidol-(4β-8)-catechin and robinetinidol-(4β-8)-catechin. The robinetinidol-(4β-8)-catechin isolated was found to be a new natural product [36].

As monomeric flavonoid compounds, wattle tannin from *A. mearnsii* bark contains leucofisetinidin, leucorobinetinidin, quercetin, myricetin, butin, butein, robtein, fisetinidol, robinetinidol, catechin, gallocatechin, fustin, dihydrorobinetin, fisetin and robinetin [37]. In addition, it contains as carbohydrates: pinitol, sucrose, glucose and fructose, and the amino acids pipecolic acid, 4-hydroxypipecolic acid, albizzine, proline, α-alanine, arginine, aspartic acid, glutamic acid and serine. A “steroid” alcohol, and a long-chain β-diketone have also been identified [38].

According to a recent phytochemical study, fractionation of *A. mearnsii* bark extract using a Diaion HP20SS column with water showed that the tannin consisted of 20.6% sugars and 72.4% polyflavonoid compounds from which sixteen compounds, including a new flavan-3-ol glycoside, 4′-*O*-methylrobinetinidol 3′-*O*-β-d-glucopyranoside and two new proanthocyanidin dimmers, fisetinidol-(4α-6)-gallocatechin and epirobinetinidol-(4β-8)-catechin were isolated and identified. In addition, the compounds robinetinidol, syringic acid, gallocatechin, catechin, taxifolin, butin, robinetinidol-(4α-8)-gallocatechin, robinetinidol-(4α-8)-catechin, fisetinidol-(4α-8)-catechin, 1,6-di-*O*-galloyl-β-d-glucose, 4-hydroxy-2-methoxyphenyl 1-*O*-β-d-glucopyranoside, 3,5-dimethoxy-4-hydroxybenzyl alcohol 4-*O*-β-d-glucopyranoside and multifidol glucoside were identified [39].

In order to determine the chemical structures of wattle tannin, small amounts (150–200 µg) of catechin, epicatechin, gallocatechin, catechin-(4α-8)-catechin and robinetinidol-(4α-8)-catechin were analyzed by pyrolysis/gas chromatography (GC). The results using this method established that pyrolysis/gas chromatography can give a rapid analysis of the degradation products. An acetone-water (70%) soluble condensed tannin extract from *A. mearnsii* bark was analyzed by using this pyrolysis/GC method and resorcinol and pyrogallol were found to be the main pyrolysis products with relatively small amounts of catechol, 4-methylcatechol and 5-methylpyrogallol. The ratio (P/C: mol/mol) of pyrogallol type B-ring to catechol type B-ring was found to be 4.21, which was consistent with that previously reported using the NMR method [40].

Since electrospray ionization (ESI) provides more reliable information on smaller molecules than matrix-assisted laser desorption ionization (MALDI) and also permits product and precursor ion investigations, a commercial wattle tannin from *A. mearnsii* bark was analyzed using a QTRAP 3200 triple-quadrupole mass spectrometer, coupled with an ESI source and the chemical composition of its proantocyanidins were determined. A total of 90.6% of the tannin extract was found to be proanthocyanidins, in which the compositions of dimers, trimers and tetramers are 42%, 40% and 8.6%, respectively. In addition, the analysis was able to provide detailed chemical structures of these proanthocyanidins [41].

Although ^1^H- and ^13^C-NMR spectroscopy has been used to elucidate the chemical structures of proanthocyanidins from bark extracts of *A. mearnsii*, particularly two-dimensional ^13^C, ^1^H-correlated (HSQC: Heteronuclear Single Quantum Correlation) spectroscopy, a new analytical method using ^31^P-NMR has recently been developed for the quantitative determination of wattle tannin from *A. mearnsii* bark. All labile hydrogens (aliphatic, phenolic hydroxys and carboxylic acid hydroxyl groups) of a commercial wattle tannin were labeled with a phosphorus-containing reagent, 2-chloro-4,4,5,5-tetramethyl-1,3,2dioxaphospholane (Cl-TMDP) and were analyzed by quantitative ^31^P-NMR and HSQC spectroscopy. The results showed that the ratio of pyrogallol to catechol was 6.8 to 1 in B-ring, whilst the A-ring substitution showed a phlorogrucinol to resorcinol ratio of 3 to 2. However, the calculated proanthocyanidins content of the sample was only 49% [42].

## 4. Biological Activities of Wattle Tannin

### 4.1. Antioxidant

The antioxidant activity of wattle tannin from *A. mearnsii* bark was first discovered in 2001 and reported in a patent publication in 2004 [1] but scientific papers on wattle tannin and its antioxidant activity have been published more recently.

In 2007, Liu et al. [43] reported a relationship between antioxidant activity of *A. mearnsii* bark extract and anti-tumor activity. Radical scavenging ability assays indicated that the proanthocyanidins crude products from the extract had a strong antioxidant activity. An anti-tumor test of the proanthocyanidins on human cancer cells cultured in vitro showed that the proanthocyanidins had a medium effect on promyelocytic cell line, weak effect on human stomach adenocarcinoma and human hepatocellular carcinoma. In 2010, Shen et al. [44] showed that free-radical scavenging activity of the proanthocyanidins from the bark of *A. mearnsii* was measured by the DPPH method and found that the proanthocyanidins product had a strong radical scavenging ability. In addition, it was also found that the proanthocyanidins had better anti-tumor activities than those from the bark of *Pinus massoniana*, and interestingly, the proanthocyanidins obtained from the ethyl acetate fraction of the wattle tannin had better anti-tumor activities than those from the water fraction. Huang et al. [45] reported also in 2010 on a relationship between antioxidant activity of *A. mearnsii* extract and viability of human neuroblastoma SH-SY5Y cells and indicated that a galloyl dimer prorobinetinidin from *A. mearnsii* De Wild. robinetinidol-(4β-8)-epigallocatechin 3-*O*-gallate (REO), has antioxidant properties and could protect the brain against acrolein-induced oxidative damage. The REO protects neurons from the deleterious effects of acrolein via the attenuation of oxidative stress. In 2017, a study on the comparison of the antioxidant activity between proanthocyanidins’ dimer from *A. mearnsii* De Wild. bark powders and catechins was made and showed that the antioxidant activity of the proanthocyanidin, procyandin-(4,6)-prorobinetinidin was higher than that of catechin and epigallocatechin gallate (EGCG) [46].

In 2018, Ikarashi et al. [47] reported the anti-hypertensive effects of the extracts from bark of *A. mearnsii.* Spontaneously hypertensive rats (SHR) with hypertension and control Wistar Kyoto rats (WKY) were fed food containing the extracts or control food for 4 weeks. The systolic blood pressure of the SHR treated with the extracts for 4 weeks were found to have decreased from the first week of treatment when compared to the systolic blood pressure of the controls. The decrease depended on the dose of the extracts. Diastolic blood pressure showed similar results. Additionally, blood SOD activity in SHR was significantly higher in the extracts group than in the control group. The anti-hypertensive effects of the extracts may be related to the anti-oxidative effects of increased blood SOD activity.

In 2004, the first patent entitled “Active oxygen scavenger prepared from Acacia plant bark, and composition made from the same” was published [48]. The superoxide sucavenging activity (SOSA) values of hot water extract from *A. mearnsii bark*, both ethanol and methanol soluble fractions from the hot water extract, vitamin C, and catechin were found to be 1900, 2400, 2100, 360, and 340 (×10^3^ unit/g), respectively. It suggested that the antioxidant activity of the extract from *A. mearnsii* was extremely high. A patent was applied for in 2006 entitled “Antioxidant composition containing component originating in the bark of tree belonging to the genus *Acacia*” [49] and another patent relating to antitumor applications in 2006 entitled “Composition for preventing and/or treating tumor containing component originating in the bark of tree belonging to the genus *Acacia*” was applied for [50]. Then in 2009, a patent entitled “Anti-oxidant compositions” was published. The specification of the patent described that an extract from the bark of *A. mearnsii* may be used as an anti-oxidant in animal feeds and in the raw materials of feeds, as well as in the prevention of the oxidation and depletion of vitamins therein and in vivo [51].

### 4.2. Antimicrobial Activity

It has been reputed that *A. mearnsii* bark extract have been used as a medicinal plant for the treatment of microbial infections in Africa. Antimicrobial effects of wattle tannin from *A. mearnsii* bark have been studied and the results are summarized in Table 1.

#### 4.2.1. Antifungal Activity

In 1994, Ohara et al. [52] studied the effects of 70% acetone/water extract from *A. mearnsii* bark on wood rotting fungi. Results of antifungal activities of the ethyl acetate-soluble fraction (EA) and water- soluble fraction (AW) on white rot fungus, *Coriolus versicolor*, showed that AW had no antifungal activity at 0.01–0.25% concentrations, and EA had very mild activities at 0.1–0.25% concentrations. Similar tendencies have been observed with brown rot fungus, *Tyromyces palustris*.

The antimicrobial activity of a crude acetone extract of *A. mearnsii* stem bark was also evaluated. Fungal isolates are shown in Table 1. The Minimum Inhibitory Concentration (MIC) values for fungal isolates were 625–5000 μg/mL. The antibiosis determination showed that the extract was more fungicidal (66.67%) than fungistatic (33.33%) [53]. Cristiane et al. [54] reported that *A. mearnsii* extract tannin showed antimicrobial activity. Inhibition activities of *Aspergillus niger* ATCC 9642 (fungus) and *Candida sp.* ATCC 14053 (yeast) were weak.

The toxicity of the tannin extract from *A. mearnsii* bark was also evaluated with *Saccharomyces cerevisiae*, Wild-type strain BY4741 and *Δgsh1* strain which lacks the glutathione enzyme. In the poisoner quantitative drop test, both strains showed growth at concentrations of 2.10 mg/L, which is comparable with the control (0.0 mg/L). However, it was observed that toxic effects on the both strains became apparent at a concentration of 4.20 mg/L. This result showed that *A. mearnsii* extract could be toxic on yeast if the extract concentrations were sufficiently high [55].

A Brazilian patent [56] filed in 2007 entitled “Antifungal composition based on plant extracts for the treatment of green wood” describes the antifungal composition which comprises 10–70% tannins: extracts from *A. mearnsii*, *Quebracho colorado*, *Caesalpinia spinosa*, *Caesalpinia coriaria*, *Rhus coriaria*, etc. and 5–30% anionic surfactant prepared in situ by neutralization of sulfonic acids with agents in aqueous solution. A Chinese patent specification [57] filed in 2007 entitled “Compounded bacteriostatic agent containing plant polyphenol” also describes agents composed of tannins from valonia, larch and/or *A. mearnsii*, *Radix glycyrrhizae* powder, *Ginkgo biloba* leaf powder and baicalein.

#### 4.2.2. Antibacterial Activity

Smith et al. [58] reported that the growth of *Escherichia coli* was inhibited by wattle tannin extract only when the tannins were exposed to oxygen. This is bacause tannins autoxidize and a substantial amount of hydrogen peroxide is generated when they are added to an aerobic medium.

Zoetendal et al. [59] found that when *E. coli* was grown with tannin extract from *A. mearnsii* bark under anaerobic conditions, its growth was not inhibited. One of the mechanisms whereby the cells of gram-negative bacteria was protected was believed to be related to genes such as the cell envelope stress protein gene *spy* and the multidrug transporter-encoding operon *mdt*ABCD, both of which are controlled by the BaeSR two-component regulatory system. Since the growth of *E. coli* mutant, which lacks the BaeSR system, was found to be inhibited by wattle tannin under anaerobic conditions, the BaeSR system may also play a prominent role.

Scientific validation of the antifungal and antibacterial activities have been reported, and these results support the use of *A. mearnsii* in traditional medicine for the treatment of microbial infections [53,60]. Olajuyigbe et al. [60] found antibacterial potentials of crude methanolic extract of the stem bark of *A. mearnsii* against some bacteria of clinical importance in shigellosis. Bacteria used in the study are shown in Table 1. The MIC values for gram-negative bacteria were 0.0391–0.3125 mg/mL while for Gram-positive bacteria they were 0.0781–0.625 mg/mL. The antimicrobial activity of a crude acetone extract of *A. mearnsii* stem bark was also evaluated. Bacterial isolates are shown in Table 1. The MIC values for Gram-positive bacteria were 78.1–312.5 μg/mL and for Gram-negative bacteria 39.1–625 μg/mL. The bacteria were more susceptible to the tannins than the fungal strains tested. The antibiosis determination showed that the extract was more (75%) bactericidal than bacteriostatic (25%) [53]. The results also showed that there was no significant differences between the MIC values of methanol extract and those of acetone extract against Gram-negative and Gram-positive bacteria.

The cytotoxicity activity of an acetone extract was observed between the concentration range 31.25 μg/mL and 500 μg/mL. The LC_50_ value 112.36 μg/mL indicated that the extract was nontoxic in the brine shrimp lethality assay (LC_50_ > 100 μg/mL) [53].

A number of additional investigations on the antimicrobial activity of tannin extracts has been reported. Cristiane et al. [54] reported that *A. mearnsii* extract tannin showed antimicrobial activity. Inhibition activities of *Staphylococcus aureus* (gram-positive) were strong. Tannins from *A. mearnsii* were encapsulated using sol-gel methods silicate route, and the hybrid materials had good antimicrobial activities, which were similar to those exhibited by the neat tannins.

The influences of acetone, methanol and aqueous extracts of *A. mearnsii* on the ultrastructures, protein and lipid leakages of five different bacteria were investigated. The bacteria used in the study are shown in Table 1. The extracts caused significant ultrastructural changes, protein and lipid leakages. While an aqueous extract was the most effective in causing protein leakages, the methanol extract was the leading cause of lipid leakages [61].

Interactions between the methanol extract of *A. mearnsii* bark and eight antibiotics were investigated by MIC, agar diffusion and checkerboard assays. Bacteria used in the study are shown in Table 1. The MICs of all the antibiotics ranged between 0.020 and 500 μg/mL while that of the the extract varied between 0.156 and 1.25 mg/mL. The checkerboard assays showed synergistic interaction (61.25%), additivity/indifference (23.75%) and antagonistic (15%) effects. Differences in the resultant synergistic, indifferent and antagonistic interactions observed in this test were due to the elevated MIC values obtained from the resistance of these bacteria to some of the antibiotics [62]. The antibacterial activities of the acetone extract from *A. mearnsii* bark and its interactions with antibiotics against some resistant bacterial strains were evaluated. The bacteria used in the study are shown in Table 1. The antibacterial combinations were mainly antagonistic than synergistic in the agar diffusion assay. Although the antibacterial combinations in agar diffusion assay were mostly antagonistic interactions, the microbroth dilution assay showed the extract and the antibiotics exerted a variable degree of inhibitory effects on the test organisms. The in vitro antibacterial activities of these antibiotics and their combinations were further assessed with the checkerboard assay to determine the fractional inhibitory concentration (FIC) index. From the checkerboard assay, the antibacterial combinations showed a variety of degrees of interactions including synergism, additive, indifference and antagonism interactions. While antagonistic and additive interactions were 14.44%, indifference interaction was 22.22% and synergistic interaction was 37.78% of the antibacterial combinations against the test isolates [63]. 

Commercially available tannin-based products were studied as natural sanitizers. Lettuce is often involved in foodborne outbreaks caused by pathogenic *E. coli.* Klug et al. [64] compared the efficacy of tannin extracts and chlorine treatments on the reduction of *E. coli* ATCC 25922 adhering to lettuce leaves. The treatment with tannin extracts from black wattle (1%, *v*/*v*) reduced the *E. coli* adhering to and under any biofilm formation on lettuce leaves and its effect was similar to that found with chlorine solutions.

#### 4.2.3. Inhibitory Effects on Cyanobacteria 

Algal bloom control by black wattle extract has been studied in China. Zhou et al. [65] showed that in a short-term test, 3–4 mg/L black wattle extract inhibited the growth of *Microcystis aeruginosa* and reduced the algal cell density in one week, whereas serious algal blooms occurred in the untreated control mesocosm. More importantly, a long-term test suggested that black wattle extract played a significant role in plankton structure optimization at the lower concentrations of 1–2 mg/L. This study provided a novel natural plant agent, which not only had positive effects on algal bloom control but also restored the aquatic ecosystem. Luo et al. [66] indicated that *A. mearnsii* extracts increased the membrane permeability of *M. aeruginosa* by damaging the ultrastructure of the algal cells, leading to a decrease in the number of algal cells and chlorophyll-a. Liu et al. [67] suggested that both the photosynthetic systems and membranes of *M. aeruginosa* were potentially damaged by the *A. mearnsii* extract. That *A. mearnsii* extract can significantly increase cell membrane permeability and Ca^2+^/Mg^2+^-ATPase activity on the membrane was demonstrated. A long-term exposure of *A. mearnsii* extract at 20 ppm led to algal cell membrane leakage or even lysis. A comparison of expression of three photosynthesis-related genes (*rbc*L, *psa*B and *psb*D) in *M. aeruginosa* with and without plant extract treatment revealed that their expression was remarkably reduced in the presence of the extract. This could indicate the inhibition of the photosynthetic process.

### 4.3. Inhibition of Enzymes

The protein-adsorbing capacities of various kinds of tannin fractions can be determined by the formation of precipitates with bovine serum albumin. Ohara et al. [53] indicated that the protein-adsorbing capacities of low-molecular weight proanthocyanidins were weaker than those of proanthocyanidin polymers. Since enzymes are protein molecules in cells, proanthocyanidins polymers are very likely able to adsorb these proteins resulting in the deactivation of the enzymes. Several studies on the enzyme inhibitions of *A. mearnsii* extract have been reported.

Takagi et al. reported the tyrosinase inhibition effects of aqueous acetone (70%) extracts of *A. mearnsii* bark, which showed high flavanol contents and strong tyrosinase inhibition, whilst the quebracho extracts inhibited tyrosinase activity only slightly despite its high flavanol content. The relation between the phenolic hydroxylation pattern and tyrosinase inhibition suggested that the proanthocyanidins with a 5,7-dihydroxyphenyl structure in the A-ring and a 3,4,5-trihydroxyphenyl structure in the B-ring have potent tyrosinase inhibitory activity [68]. Before these results were reported in the scientific journal in 2003, a patent entitled “Preparation for external use for skin/Novel use of extract isolated from bark of larch, acacia or duramen of *Schinopsis lorentsii* as skin whitening agent” was published in 1998 [69]. The patent describes that an extract from bark of *A. mearnsii* has an inhibition effect on the tyrosinase activity and can be used for producing an external preparation for skin whitening. More recently, a Chinese patent “Skin deep-clean facial cream for suppressing tyrosinase and decomposing melanin” was published in 2017 [70]. The title facial cream comprises (by wt %): *A. mearnsii* bark proanthocyanidins 0.01–0.5%.

Other studies on the inhibition of digestive enzymes such as lipase, α-amylase and glucosidase have been reported. Kusano et al. [40] indicated that the bark extract from *A. mearnsii* showed strong lipase and α-amylase inhibition. Active substances responsible for the inhibition were found to be proanthocyanidins oligomers, which are mainly composed of 5-deoxyflavan-3-ol units with pyrogallol- and catechol-type B-rings. Mariano et al. [71] showed that a condensed tannin from *A. mearnsii* was an effective inhibitor of both human salivary and porcine pancreatic α-amylase. Similarly, the extract from pinhão (*Araucaria angustifolia*) seed coat was also effective in diminishing the post-prandial glycemic levels in rats after starch administration. Matsuo et al. [72] showed that the extent of the strength of α-amylase inhibitory activity depended on the B-ring’s structures of the proanthocyanidins. The spectroscopic results clearly indicated that fractions with strong inhibitory activity contained proanthocyanidins oligomers with catechol-type B-rings rather than pyrogallol-type B-rings. HPLC analysis of the pyrolysis products showed peaks for pyrocatechol were only observed in the mixtures obtained from the fractions with high inhibitory activities. Kato et al. [73] indicated that the human salivary α-amylase was more strongly inhibited by hydrolysable tannin than by condensed tannin with the concentrations for 50% inhibitory concentration (IC_50_) being 47.0 and 285.4 μM, respectively. The inhibitory capacities of both tannins on the pancreatic α-amylase were also different, with IC_50_ values being 141.1 μM for the hydrolysable tannin and 248.1 μM for the condensed tannin. Xiong et al. [74] compared the anti-inflammatory and digestive enzymes (α-glucosidase and α-amylase) inhibitory activities of the polyphenols isolated from *A. mearnsii* bark crude extract and fractions (Fr.1–Fr.7) obtained by high-speed counter-current chromatography (HSCCC). Fractions B4, B5, B6, B7 (total phenolics 850.3, 983.0, 843.9, and 572.5 mg/g, respectively) showed significant activities against reactive oxygen species (ROS), nitric oxide (NO) production, and expression of pro-inflammatory genes interleukin-1β (IL-1β) and inducible nitric oxide synthase (iNOS) in a lipopolysaccharide-stimulated mouse macrophage cell line RAW 264.7 (a mouse macrophage cell line ATCC TIB-71). All of the crude extract and the fractions suppressed α-glucosidase and α-amylase activities. 

### 4.4. Anti-Obesity and Anti-Diabetes

Wattle tannin from *A. mearnsii* bark has been studied as a functional substance. Ikarashi et al. [75] investigated the anti-obesity/anti-diabetic effects of the extracts from the bark of *A. mearnsii* using obese diabetic KKAy mice. Increases in body weight, plasma glucose and insulin were significantly suppressed for the extract groups. The mRNA expression of energy expenditure-related genes (PPARα, PPARδ, CPT1, ACO and UCP3) in skeletal muscle increased, and the protein expressions of CPT1, ACO and UCP3 also increased. However, the expression of fat acid synthesis-related genes (SREBP-1c, ACC and FAS) in the liver decreased. The mRNA expression of adiponectin increased while the TNF-α in white adipose tissue decreased. These results indicated that the anti-obesity actions of the extract of *A. mearnsii* bark are attributable to increased expression of energy expenditure-related genes in skeletal muscle, and decreased fatty acid synthesis and fat intake in the liver.

Another mechanism for the anti-obesity and anti-diabetes, reduction in the intestinal absorption of lipids and carbohydrates has been reported by Ikarashi et al. [76]. In an in vitro study, the inhibitory activity of extracts from the bark of *A. mearnsii* on lipase and glucosidase was measured. The effects of the extracts on absorption of orally administered olive oil, glucose, maltose, sucrose and starch solution in mice were evaluated. The concentration of the extracts were found to inhibit the activity of lipase, maltase and sucrase with IC_50_s of 0.95, 0.22 and 0.60 mg/mL, respectively. When oral administration of the extract solutions was used on ICR mice, the extracts significantly inhibited the increase in plasma triglyceride concentration after olive oil loading. The extracts also significantly inhibited the increase in plasma glucose concentration after maltose, sucrose or glucose loading. These results suggested that the extracts inhibited lipase and glucosidase activities, which lead to a reduction in the intestinal absorption of lipids and carbohydrates. The inhibition of glucose uptake via a sodium-dependent glucose transporter (SGLT) and glucose transporter (GLUT) may be attributable to the extracts. 

Before the use of proanthocyanidins as anti-obesity and anti-diabetes materials was reported in the scientific literature, two patents were filed. The first was published in 2006 [77] entitled “Hypoglycemic composition containing component originating in the bark of tree belonging to the genus *Acacia*”, while the other entitled “An anti-obesity composition extracted from bark of trees belonging to *Acacia*” was filed in 2006 [78]. This patent specification describes polyphenols from bark of *A. mearnsii* De Wild. having effects in preventing and treating obesity, hypertension, diabetes, fatty liver, arteriosclerosis, cerebral infarction, hyperlipidemia, peripheral blood vessel dysfunction and ischemic heart disease, and the polyphenols could be incorporated into food, animal feed or pharmaceuticals, or used as an external use medicine.

### 4.5. Other Biological Activities

The aqueous-soluble fraction (AW) and the ethyl acetate-soluble fraction (EA) from the 70% acetone-water soluble extract from *A. mearnsii* bark described in Section 4.2.1 were considered to be promising naturally occurring termiticides because they had the ability to deactivate some insect enzymes. Results from termite tests showed that the EA and AW had remarkable anti-termite activities. Therefore, Ohara et al. [54] suggested that toxicities of proanthocyanidins as phenolic compounds might be involved in the anti-termite activities of the substances. In 2006, a patent [79] entitled “Aqueous extract to repel or exterminate termites” was published. The invention was aimed at the development of an aqueous solution based on a modified aqueous vegetable extract of tannins that could repel or exterminate termites. Active ingredients were based on aqueous organic sources such as tannins from the black wattle tree (*A. mearnsii* De Wild). In 2008, another patent entitled “*Acacia* mearnsii bark extract as insecticide” was published. The extract from *A. mearnsii* bark had been found to be an insecticide suitable for the control of *Aedes aegypti*, *Culex quinquefasciatus* and *Simulium pertinax* [80].

In 2012, the effect of the extract from *A. mearnsii* bark on skin was studied. By using HR-1 mice with atopic dermatitis (AD), an improvement in atopic dermatitis symptoms was observed when the mice were fed the extract. A ceramide expression in the skin was not changed in the *Acacia* group despite a decrease in the AD group. The mRNA expression of ceramidase was found to decrease in the *Acacia* group compared to the AD group. The extract from *A. mearnsii* bark appeared to inhibit itching in atopic dermatitis by preventing the skin from drying. The mechanism by which this occurred involved the inhibition of increased ceramidase expression associated with atopic dermatitis [81]. In 2001, a patent entitled “Histamine liberation suppressing agent/Histamine release inhibitor containing plant extracts for relieving inflammation and preventing periodontitis” was filed. The patent specification described a safe histamine release inhibitor comprising wattle bark (bark of *A. mearnsii*). This could be formulated into cosmetics and pharmaceuticals for relieving inflammation, or formulated into dentifrice or oral drug for preventing periodontitis [82]. In 2006, the patent “Composition for preventing and/or treating itching containing component originating in the bark” was published [83]. 

Xiong et al. [74] showed that polyphenols could be isolated from a crude extract of *A. mearnsii* bark and evaluated their anti-inflammatory effects. The study showed that the crude extract could significantly decrease the non-mitochondrial oxidative burst that is often associated with an inflammatory response in human monocytic macrophages.

## 5. Conclusions

Even though the name of the genus *Acacia* was derived from *Acacia nilotica*, the name of the species originally known as *Acacia nilotica* was recently changed to *Vachellia nilotica*, whilst the name of *Acacia mearnsii* has remained.

In order to determine the amount of active compounds in wattle tannin for a specific application, a number of analytical methods are in use. When tannin is to be used for the production of a wood adhesive, the Stiasny method is used; however when the tannin is to be used in the tanning of hides, the hide-powder method is usedA simple UV method has been found to estimate values found using both the Stiasny and hide-powder methods. The tannin contents in bark can also be estimated using NIR and NMR, although these techniques require data from wet chemistry.

The chemical composition of wattle tannin has been determined using traditional organic chemistry techniques such as isolation and identification of compounds. Tannin consists of polyflavanoids whose molecular masses which may range from 300 to 3000 with flavanoid units such as fisetinidol, robinetinidol, catechin and gallocatechin. All the chemical compounds isolated from wattle tannin have been updated. More detailed chemical composition information can be obtained using pyrolysis/GC analysis, electrospray mass spectrometry investigation and quantitative ^31^P-NMR and HSQC analyses.

Studies on the relationships between antioxidant and anti-tumor activity of wattle tannin, viability of human neuroblastoma SH-SY5Y cells and the anti-hypertensive effects have been studied. Robinetinidol-(4β-8)-epigallocatechin 3-*O*-gallate was found to have antioxidant properties and could protect brain cells against acrolein-induced oxidative damage.

The antioxidant activity of proanthocyanidins from tannin was higher than that of flavan-3-ols monomers such as catechin and epigallocatechin gallate. As there are many reports on the relationship between antioxidant and a number of biological activities, the possibilities exist for making use of these properties of proanthocyanidins.

A total of fourteen papers and two patents reported the antimicrobial activities of wattle tannin. Generally, bacteria were more susceptible to the tannins than the fungal strains tested. Several bacteria were inhibited by the extract from *A. mearnsii* bark. The growth inhibition mechanisms of *E. coli* were investigated. An interaction between extracts from *A. mearnsii* bark and antibiotics have also been studied. The extracts from *A. mearnsii* bark inhibit the growth of cyanobacteria. 

The proanthocyanidins from wattle tannin have the ability to inactivate digestive enzymes such as α-amylase, lipase and glucosidase. In vivo experiments on anti-obesity and anti-diabetes have also been reported. Several patents describing the behavior of these digestive enzymes in anti-diabetes and anti-obesity studies have been reported. There have been reports on the use of *Acacia* bark extract as an antitermite material, on the inhibition of itching in atopic dermatitis and anti-inflammatory effects. 

In conclution, the growth of bacteria was inhibited by the extract from *A. mearnsii* bark, and a typical intestinal bacterium such as *E. coli*, *K. pneumoniae*, *P. vulgaris* and *S. marcescenes* was also inhibited by extracts. Based on these results, the *Acacia* bark extract may inhibit not only the growth of *E. coli*, *K. pneumoniae*, *P. vulgaris* and *S. marcescenes* but also the growth of other types of intestinal bacteria such as *Clostridium* and *Bacteroides*, the so-called “bad bacteria” If tannin extract from *A. mearnsii* bark inhibits growth of these “bad bacteria” in vivo evaluation, the extracts might be able to be used as a new dietary supplement, which could control the human intestinal microbiome to keep the body healthy.

## Figures and Tables

**Table 1:** Antimicrobial effects of wattle tannin from *A. mearnsii* bark.

No.	Extracts	Tested Bacterial Isolates	Growth or Inhibition	Reference
1 *	Water soluble fraction (AW) from 70% acetone-water soluble extract	*Coriolus versicolor* *Tyromyces palustris*	No antifungal activity at 0.01–0.25% concentrations	[52]
	Ethyl acetate soluble fraction (EA) from 70% acetone-water soluble extract	*C. versicolor* *T. Palustris*	Very mild activities at 0.1–0.25% concentration	
	Crude acetone extract	*Candida krusei* *Candida albicans* *Candida rugosa* *Candida glabrata* ATCC 2001 *Absidia corymbifera* *Fusarium sporotrichioides* *Trichophyton tonsurans* *Trichophyton mucoides* ATCC 201382 *Aspergillus niger* *Aspergillus terreus* *Aspergillus flavus*	MIC values were fungal isolates (625–5000) μg/mL	[53]
	Tannins	*A. niger* ATCC 9642 *Candida* sp. ATCC 14053	Weak inhibition activity	[54]
2 *	Aqueous extract	*Saccharomyces cerevisiae* BY4741 *S. cerevisiae* Δ gsh1	In the poisoner quantitative drop test, toxicological effects from a concentration of 4.20 mg/L	[55]
	Crude acetone extract	*Escherichia coli* ATCC 25922 *Bacillus cereus* ATCC 10702 *Proteus vulgaris* KZN *Serratia marcescens* ATCC 9986 *Pseudomonas aeruginosa* ATCC 19582 *Enterococcus faecalis* KZN *Klebsiella pneumoniae* ATCC 10031 *P. vulgaris* CSIR 0030 *Bacillus pumilus* ATCC 14884 *K. pneumoniae* KZN *Staphylococcus aureus* OK1 *Salmonella typhi* ATCC 13311	MIC values were Gram-positive bacteria (78.1–312.5) μg/mL, Gram-negative bacteria (39.1–625) μg/mL	[53]
	Tannins	*S. aureus*	Strong inhibition activity	[54]
	Aqueous extract	*E. coli* BW13711 *E. coli* TA4131	No growth in 0.1% wattle tannin extract medium	[58]
		*E. coli* WTT1 *E. coli* TA4110	No growth in 0.15% wattle tannin extract medium	
	Aqueous extract	*E. coli* BW13711 *E. coli ΔBae*SR13711	Growth of the *E. coli Δ*BaeSR mutant reached behind stationary phase compared to that of *E. coli* BW13711 in the presence of tannins	[59]
	Crude methanol extract	*E. coli* ATCC 8739 *K. pneumoniae* ATCC 10031 *B. pumilus* ATCC 14884 *P. vulgaris* ATCC 6830 *Acinetobacter calcoaceticus* *A. calcoaceticus* anitratis CSIR *P. vulgaris* CSIR 0030 *Shigella flexneri* KZN *S. typhi* ATCC 13311 *Micrococcus luteus* *E. faecalis* KZN *S. aureus* OK2b *S. aureus* OK1 *S. aureus* OK3	Minimum inhibitory concentration (MIC) values were gram-negative (0.0391–0.3125) mg/mL and gram-positive bacteria (0.0781–0.625) mg/mL	[60]
	Acetone, methanol and aqueous extracts	*E. coli* *S. aureus* *B. pumilus* *P. vulgaris* *S. flexneri*	Extracts caused the disruption of the cytoplasmic membranes of the bacterial cells.	[61]
	Methanol extract	*S. aureus* ATCC 6538 *E. faecalis* ATCC 29212 *E. faecalis* ATCC 29212 *E. coli* ATCC 25922 *Bacillus subtilis* KZN *P. vulgaris* KZN *E. faecalis* KZN *Enterobacter cloacae* ATCC 13047 *K. pneumoniae* (ATCC 10031) *P. vulgaris* ATCC 6830 *Shigella sonnei* ATCC 29930	Synergetic, indifferent and antagonistic interactions were differences depending on combination between bacterial types and antibiotics agent types with the extract	[62]
3 *	Acetone extract	*E. coli* ATCC 25922 *B. cereus* ATCC 10702 *P. aeruginosa* ATCC 19582 *S. marcescens* ATCC 9986 *E. faecalis* KZN *S. aureus*_ok1_ *S. flexneri* KZN *M. luteus* *P. vulgaris* CSIR 0030 *S. typhi* ATCC 13311	Synergetic, additive, indifference and antagonism interactions were differences depending on combination between bacterial types and antibiotics agent types with the extract	[63]
	Tannin extract	*E. coli* ATCC 25922	Tannin extract is capable of reducing the counts of *E. coli* adhered to and under biofilm formation on lettuce leaves.	[64]
	Aqueous extract	*Microcystis aeruginosa*	Black wattle extract inhibits algal blooms in a short-term test and the extract maintains water quality and prevents algal blooms in a long-term test.	[65]
	Extract	*M. aeruginosa*	Extract damage to the ultrastructure of the algal cell and decrease algal cells and chlorophyll-a.	[66]
	Aqueous extract	*M. aeruginosa*	Expression of photosynthesis-related genes was remarkably reduced in the presence of the extract.	[67]

1 *: Antifungal activity, 2 *: Antibacterial activity, 3 *: Inhibitory effect on cyanobacteria.
